# An enriched sugarcane diversity panel for utilization in genetic improvement of sugarcane

**DOI:** 10.1038/s41598-020-70292-8

**Published:** 2020-08-07

**Authors:** Nathanael D. Fickett, Leila Ebrahimi, Arnold P. Parco, Andres V. Gutierrez, Anna L. Hale, Michael J. Pontif, James Todd, Collins A. Kimbeng, Jeffrey W. Hoy, Tomas Ayala-Silva, Kenneth A. Gravois, Niranjan Baisakh

**Affiliations:** 1 School of Plant, Environmental and Soil Sciences, Louisiana State University Agricultural Center, Baton Rouge, LA 70803 USA; 2 Sugarcane Research Unit, USDA-ARS, Houma, LA USA; 3 Sugar Research Station, Louisiana State University Agricultural Center, St. Gabriel, LA USA; 4 Department of Plant Pathology and Crop Physiology, Louisiana State University Agricultural Center, Baton Rouge, LA USA; 5 Tropical Agricultural Research Station, Mayaguez, PR USA; 6 Present Address: Vermont Mutual Insurance Group, Montpelier, VT USA; 7 Present Address: University of Tehran, Aburaihan Campus, Tehran, Iran; 8 Present Address: Certis Inc., Baton Rouge, LA USA

**Keywords:** Natural variation in plants, Plant breeding, Plant genetics

## Abstract

Sugarcane crop is important for both sugar and biofuels. A world collection of sugarcane and related grasses (WCSRG) maintained at Miami, FL contains > 1,200 non-redundant clones of different species and genera within the *Saccharum* complex. However, linkage of undesirable alleles with useful genes in wild species has hindered its efficient utilization in sugarcane breeding. A core collection developed previously with smaller number of clones representing WCSRG did not take into account > 120 wild/exotic clones maintained at the USDA-ARS Sugarcane Research Unit in Houma, Louisiana. Moreover, the genome complexity and sub-tropical to temperate growing climate of Louisiana warrant a region-specific core collection that can be used for base-broadening breeding aimed at efficient introgression of desirable alleles. Genetic diversity of 1,485 clones within WCSRG and Louisiana (commercials, wild/exotic) using 423 SSR alleles showed an average gene diversity (h) at 0.208 among all species groups where *Erianthus*-like *Saccharum* species (ELSS), *Miscanthus* spp., and *S. spontaneum* each formed a distinct cluster, *Saccharum robustum, S. officinarum,* hybrid cultivars, and *S. edule* grouped together in a major cluster, and *Saccharum sinense* and *S. barberi* formed distinct grouping. A 309-clone diversity panel (SDP1) was developed that captured the genetic diversity based on the combination of maximum length subtree and manual selection to maximize representation of Louisiana clones and minimize import of clones from Miami. SDP1 shared 324 alleles out of the 423 alleles in the entire population of 1,485 clones and captured the genetic diversity of the entire collection with an average gene diversity (h) at 0.163. The variation within (11–17%) and among (83–89%) the populations in SDP1 were comparable with the entire population of 1,485 clones (9–15% and 85–91%, respectively). The breadth of the genetic variation of SDP1 was exemplified by the intra- and inter-specific diversity of a 190-clone mini-core collection with markers derived from known cold-responsive genes. SDP1 will facilitate genome-wide association studies for identification of trait-specific markers for use in marker-assisted breeding in Louisiana and elsewhere.

## Introduction

Cultivated sugarcane (*Saccharum* interspecific hybrids) is a perennial C_4_ grass crop belonging to the subtribe Saccharinae under the tribe Andropogoneae of the family Poaceae. Sugarcane is cultivated worldwide in tropical and subtropical regions as a major source of sucrose^1^, with a global crop value of $61 billion per year^2^. Sugarcane accounts for 80% of global sucrose production and 45% of sucrose production in the United States^3^. Sugarcane, in addition to its importance as a food crop, is recognized as the most productive bioenergy crop because of its ability to produce high biomass^4^.

Historically, six species are considered important in the *Saccharum* genus, which include two wild species, *S. spontaneum* (L.) (2n = 40–128) and *S. robustum* (Brandes & Jesw. Ex Gressl) (2n = 60, 80), and four domesticated species, *S. officinarum* (L.) (2n = 80), *S. sinense* (Roxburgh) (2n = 81–124), *S. barberi* (Jeswiet) (2n = 111–120), and *S. edule* (Hassk) (2n = 60–80)^5,6^. Genetic evidence suggests *S. robustum* as the progenitor of both *S. officinarum* and *S. edule*^7,8^, and that most modern cultivars, along with *S. sinense* and *S. barberi*, are interspecific hybrids between *S. officinarum* and *S. spontaneum*. Around 32–39% of the *S. sinense* and *S. barberi* genomes come from *S. spontaneum,* while for modern cultivars, the percentage is around 10–20%^6,9,10^.

Modern cultivars have limited genetic variation. Fewer than 20 *S. officinarum* clones are involved in the genealogy of sugarcane cultivars with only a few being used extensively^11^. Basic crosses are made with clones of *S. spontaneum, S. robustum,* and species of other genera within the *Saccharum* complex to broaden the genetic base^5,12^. In Louisiana, the basic breeding program utilizes wild (basic) clones as nonrecurrent parents, where selected recurrent interspecific hybrids are used for backcrossing^13,14^.

Commercial sugarcane breeding is labor-intensive and time consuming. In Louisiana, it takes 13 years from crossing to the release of a new variety. Phenotype-based trait selection in conventional breeding can be problematic because of confounding environmental effects. Therefore, accurate phenotypic selection in the early stages of breeding remains a challenge^15^. Selection in the early stages of breeding using family appraisal followed by mass selection attempts to separate the environmental components of total phenotype^16,17^, but there is typically limited improvement in the genetic gains in sugarcane primarily due to the quantitative nature of the traits with low to moderate heritability. Marker-assisted selection (MAS), on the other hand, could enhance the efficiency of early stage selection as well as selection response of difficult-to-phenotype traits^18^.

Quantitative trait locus (QTL) mapping is widely used to understand the genetics of complex polygenic traits, and it is the first step toward development of trait-based markers for use in MAS. Use of SNP genotyping by sequencing has improved the resolution of QTLs with generation of high-density linkage maps^19–21^. However, traditional biparental linkage mapping in heterozygous species, such as sugarcane, with only single-dose markers, may identify low-resolution QTLs due to the lack of information on the number/type of alleles at the segregating locus and limited genetic variation in the biparental population^22^.

Genome-wide association study (GWAS), on the other hand, has been used recently to identify marker-trait associations (MTAs) in several plants. GWAS allows identification of QTLs with resolution at the gene level, as it takes advantage of historical and evolutionary recombination events in a genetically diverse population (diversity panel)^23^. Further, a diversity panel allows study at the same time of allelic diversity and haplotypes of genes/alleles associated with traits. GWAS is based on linkage disequilibrium (LD), which is high in sugarcane^11,24^ mainly due to relatively few generations between modern cultivars and the limited number of initial clones used in hybridization^25,26^. The high LD of sugarcane has been exploited to identify markers associated with various traits using a regional diversity panel or core collection^18,27–32^.

*Saccharum* spp. and related genera from countries around the world have been collected into a “World Collection of Sugarcane and Related Grasses” (WCSRG) maintained at the National Germplasm Repository of the USDA-ARS Subtropical Horticulture Research Station, Miami, FL, USA. Various studies have described the diversity in the WCSRG^14,33–36^. A total of 342 *S. spontaneum* clones were assessed using stratified random sampling over geographical origins and principal component cluster groups to select a 75-clone core collection^33^. The authors also evaluated diversity of 32 *S. officinarum*, 30 *S. barberi*, 28 *S. sinense*, and 27 *S. robustum* based on cluster analysis using principal component analysis of sugar composition^34^. A 300-clone diversity panel was created based on genetic diversity of 1,002 clones of WCSRG using 231 SSR alleles^35^. A similar panel was developed through phenotypic characterization of the collection^14^.

The panels discussed above^14,33–35^ did not include clones outside of the world collection. For example, clones that have been procured and maintained by the base broadening (basic breeding) program at the USDA-ARS Sugarcane Research Unit in Houma, Louisiana and elite cultivars and parents used in breeding programs in Louisiana were not included in the diversity analysis. Moreover, ~ 250 additional clones have been collected into the WCSRG in the last four years^36^. Therefore, the core collections previously developed may not fully account for the range of variation among the subtropical and temperate sugarcane clones currently being used in Louisiana. Sugarcane crop grows optimally at ~ 35 °C. Cold stress by temperatures near freezing can compromise crop growth, development, and yield. Temperatures as high as 20 °C can suppress plant growth and below 15 °C can cause tissue injury^37^. Louisiana represents the far northern limit of sugarcane cultivation in the U.S. with frequent freezing during the crop season. Therefore, a *Saccharum* diversity panel developed from WCSRG and clones in Louisiana breeding programs would facilitate GWAS studies for identification of trait-specific markers, such as cold tolerance, for use in marker-assisted breeding in Louisiana and other sugarcane industries. Here, we report on the development of an inclusive sugarcane diversity panel (SDP1) and demonstrate the breadth of its diversity with regard to abiotic (cold) stress responsive genes.

## Methods

### Plant materials

**Figure 1:**
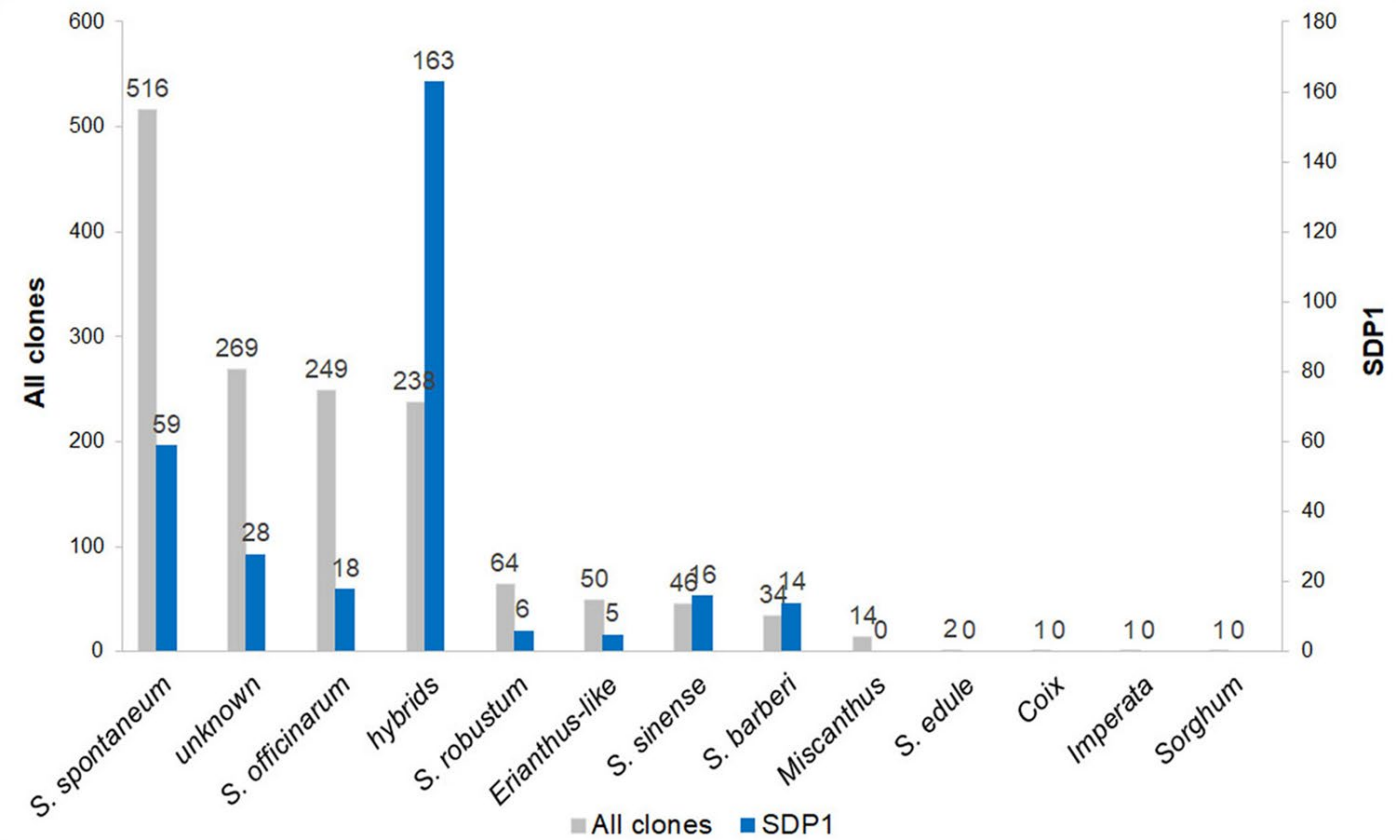
Frequency of species groups for the entire population (1,485 clones) and the diversity panel (309 clones). The two axes are proportional based on set size for comparison. *Erianthus-*like *Saccharum* spp. included *S. arundinaceum*, *S. bengalense*, *S. ravennae*, *S. rufipilum*, *S. brevibarbe, S. kanashiroi, S. procerum,* and unknown species previously identified as *Erianthus.*

For the diversity panel, 1,485 clones within the *Saccharum* complex, including *Saccharum, Miscanthus*, *Coix*, *Imperata*, and *Sorghum*, were used^36^ (Fig. 1). Of these, 1,236 were clones from the WCSRG, 113 clones were elite and historic breeding clones from the Louisiana sugarcane breeding program, 119 were clones of wild/exotic species (not present in WCSRG), and 17 were hybrids from the base-broadening introgression program of the USDA-ARS Sugarcane Research Unit. *Saccharum* spp. previously classified as *Erianthus* spp. including *S. arundinaceum*, *S. bengalense*, *S. ravennae*, *S. rufipilum*, *S. brevibarbe, S. kanashiroi,* and *S. procerum* were grouped together as *Erianthus-*like *S.* spp. (ELSS) for simplicity of analysis. For diversity analysis with cold responsive genes, a mini-core of 190 clones (Supplementary Table S1) was selected based on genetic diversity analysis of 1,485 clones where clones from each subclusters in all species groups were represented. The mini-core comprised of 96 Louisiana sugarcane elite and historic breeding clones (*Saccharum* interspecific hybrids), 27 *S. spontaneum*, 20 *S. spontaneum* (previously listed as unknown), 15 *S. officinarum*, seven *Eriranthus*, five each of *S. robustum* and *S. bengalense*, three each of *S. arundinaceum* and *Miscanthus*, two each of *S. kanashiroi* and *S. ravennae*, and one clone each of *S. edule*, *S. barberi*, *S. rufipilum*, *Coix* and *Imperata*.

### DNA purification and genotyping

Total genomic DNA was extracted from ~ 100 mg leaf tissues using the CTAB miniprep and checked for quality and quantity using a ND-100 spectrophotometer (Nanodrop Technologies Inc, Wilmington, DE), as described previously^36^. Four-hundred-fifty SSR primer pairs, including 277 genomic SSRs^38,39^, 127 eSSRs from sugarcane cold-responsive genes^40^, and 46 eSSRs from brown rust-responsive genes^41^, were initially tested for polymorphism among 113 Louisiana clones. Eleven SSR primer pairs mapped on nine out of 10 sugarcane monoploid homeologous groups^42^ and 17 out of 32 pseudochromosomes^43^ that had high polymorphism index (Supplementary Table S2) were selected to evaluate genetic diversity of the 1,485 clones.

For genetic diversity analysis of the 190-clone mini-core, 9,974 cold responsive genes reported in *Sorghum*^44^ were searched for the presence of simple sequence repeat (SSR) motifs using BatchPrimer3 v1.0 with criteria set to at least five repeats for dinucleotide motifs and three repeats for tri, tetra, penta and hexa nucleotide motifs. Primers flanking SSR motifs ≥ 16 nt from 52 genes were designed using BatchPrimer3 v1.0. In addition, 48 eSSRs from cold responsive genes of sugarcane^40^ were included.

The polymerase chain reaction (PCR) and resolution of PCR products were performed following a method described earlier^21^. Briefly, 50 ng of genomic DNA was used as the template in 10 µl PCR containing 2 µl of 5 × buffer, 1 µl of 25 mM MgCl_2_, 1 µl of 2 mM dNTP mix, 0.1 µl of Taq DNA polymerase (Promega, Madison, WI) and 0.5 μl of 10 µM forward and reverse primer (IDT, Corvallis, OR). A thermal profile of 95 °C for 5 min followed by 35 cycles of 95 °C for 15 s, 58 °C for 15 s, and 72 °C for 30 s, and 72 °C for 10 min was used. The amplification products were resolved in a 13% polyacrylamide gel using a high-efficiency gel electrophoresis system (Nihon Eido, Tokyo, Japan). Amplified fragments (alleles) were manually scored as “1” (present, dominant) and “0” (absent) in a binary matrix.

### Genetic diversity analysis

Alleles at SSR loci occurring in less than 1% of the clones were discarded prior to downstream analysis to reduce false similarity between clones due to shared absence of alleles while still capturing rare alleles. The polymorphism information content (PIC) was computed for each SSR marker following^45^.

GeneAlEx 6.502^46^ was used to compute gene diversity (h), Shannon’s information index (I), Nei’s genetic distance (D), principal coordinate analysis (PCoA), and an analysis of molecular variance (AMOVA). AMOVA was done for species groups by recorded names: *S. spontaneum, S. officinarum,* hybrid cultivar*, S. robustum,* ELSS*, S. sinense, S. barberi, Miscanthus* spp., and other^46^. AMOVA was also conducted on species groups devised from the neighbor-joining analysis, and on groups devised from the population structure analysis (described below). Private alleles, population differentiation and gene flow were estimated for the mini-core by F_ST_ and N_m_ values, respectively. For the mini-core, h and I for every locus, genetic diversity within a population (H_s_), total heterozygosity (H_t_), gene flow (N_m_), and G_st_ were calculated using PopGene 1.32^47^.

Genetic diversity was also analyzed using DARwin 6.0.12^48^ using Dice dissimilarity scores to validate the clustering of the clones. Weighted neighbor-joining algorithms were used to construct a phylogenetic tree with 1,000 bootstrap repetitions to evaluate the robustness and significance of each node. Bootstrap analysis was implemented with 1,000 iterations to compute the minimum number of SSR alleles needed to differentiate the species using Bootsie software^49^ and allele numbers were plotted against their corresponding CV values in a bi-plot curve.

### Structure analysis

Assignment of clones to a specified number of clusters (K) and population structure were determined using Structure ver. 2.3.4^50^. Models were run using Bayesian algorithm for *K* = 2—10, and *K* = 8 was selected as per the software’s documentation and eight species groups. A standard admixture model was used with an inferred alpha. To accommodate minor alleles, lambda was evaluated at different levels, and a lambda of 0.5 yielded the best models based on the log of the probability of the data. The Markov chain Monte Carlo program converged well before 50,000 iterations, so 50,000 iterations were used for ‘burn-in’, and 25,000 subsequent iterations were used for model parameter estimation. To estimate the number of clusters, an admixture model with correlated allele frequencies was run in 10 models, and two non-symmetric modes were found. One mode occurred seven times and the other was less consistent and occurred three times. An average of the seven runs from the first mode was used for the final result.

## Results

### SSR genotyping

The 11 SSR primer pairs resulted in a total of 423 polymorphic alleles (Supplementary Table S2). The number of alleles per SSR ranged from 13 to 65 with an average of 38. Three sugarcane SSRs on homeologous group (HG) 4 generated 106 alleles. All other HGs were represented by a single SSR producing 18 (HG 9) to 58 (HG 5) alleles with an average major allele frequency of 0.76. The PIC values ranged from 0.17 to 0.38 with an average of 0.25. The average number of alleles per clone-SSR pair was 8.01 with the maximum being 30.16. The maximum average number of alleles per clone for an SSR was 16.28, which is typical of sugarcane where the basic chromosome number of the species in the population ranges from x = 7 to x = 19^5^. *Saccharum spontaneum*, representing one-third of the population, has a basic chromosome number of 8.

The low frequencies of *S. sinense, S. barberi*, and *Miscanthus* spp. necessitated maintaining minor alleles. Thus, alleles with frequencies between 0.990 and 0.010 were retained for analysis. These bounds were equivalent to the frequency of *Miscanthus* spp. in the population, which was 0.009. All SSR primers produced at least one polymorphic allele with a frequency at or above 0.348, higher than the average allele frequency (0.210).

### Gene diversity and allele polymorphism by species

The gene diversity (h) ranged from 0.16 for *Miscanthus* spp. to 0.24 for *S. spontaneum* indicating that *Miscanthus* spp. were the least diverse and *S. spontaneum* the most diverse. The overall average gene diversity of all species groups was 0.21 (Supplementary Table S2). The number of accessions evaluated for each species apparently influenced the gene diversity. For example, number of *Miscanthus* accessions (14) were 37-fold less than the *S. spontaneum* (516).

The entropy measured by Shannon’s information index (I) followed the same trend with those species having higher gene diversity also exhibiting higher entropy (Supplementary Table S2). The only exceptions were *S. officinarum* and *S. barberi*, where *S. officinarum* had h = 0.21 but an I = 0.33, and *S. barberi* had an h = 0.21 but an I = 0.33. The I value ranged from 0.25 (*Miscanthus* spp.) to 0.39 for (*S. spontaneum*), with an average of 0.33.

The percentage of polymorphic alleles within a species was apparently directly proportionate to its population size (Fig. 1, Supplementary Table S3). Again, the polymorphic alleles were highest for *S. spontaneum* (99.3%) and lowest for *Miscanthus* spp. (54.4%).

### Genetic distance between species

Based on Nei’s pairwise genetic distance (D), the greatest distance was between the hybrid cultivars group and *Miscanthus* spp. at 0.105, while the shortest genetic distance was between *S. officinarum* and hybrid cultivars group (0.009), followed by the D between *S. officinarum* and *S. robustum* (0.011) (Supplementary Table S4). *Miscanthus* spp. were the farthest from the other species groups. *Miscanthus* spp. were closest to ELSS and *S. spontaneum* at D = 0.038 and D = 0.049, respectively, but farthest from *S. sinense* (D = 0.101). The D value between the rest of the species groups and *Miscanthus* spp. ranged from 0.07 to 0.105. The next farthest from the others was ELSS (0.024 to 0.086) followed by *S. spontaneum* (0.032 to 0.060).

### Phylogeny and population structure

**Figure 2:**
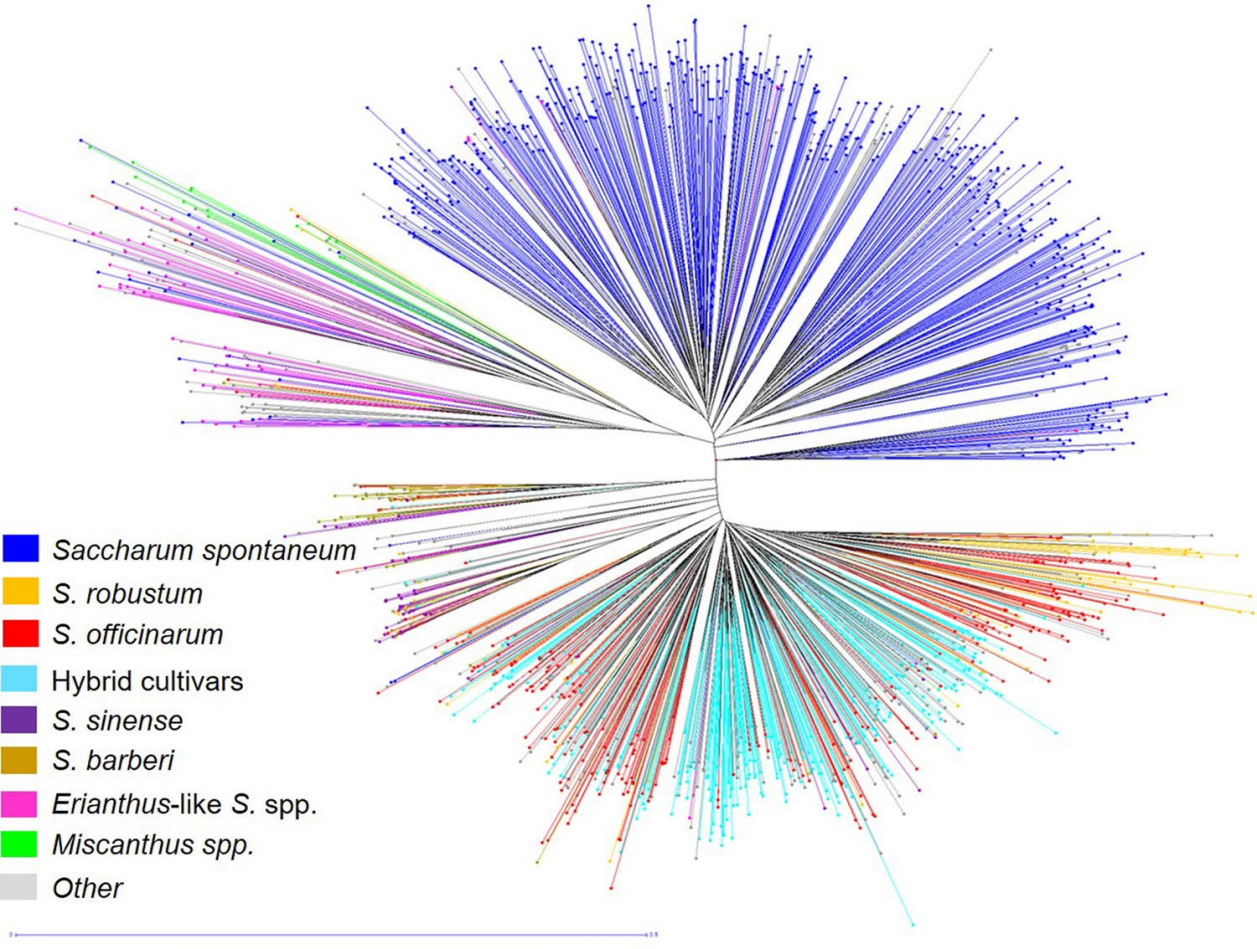
Neighbor-joining tree of the *Saccharum* complex showing genetic diversity based inter- and intra-species differentiation of 1,485 clones using 423 SSR alleles. *Erianthus-*like *Saccharum* spp. included *S. arundinaceum*, *S. bengalense*, *S. ravennae*, *S. rufipilum*, *S. brevibarbe, S. kanashiroi, S. procerum,* and unknown species previously identified as *Erianthus.* Other species included *Coix lacryma-jobi, Imperata* sp., *Sorghum polumosum, Saccharum edule,* and unknown.

Neighbor joining with a Dice dissimilarity matrix was also used to evaluate genetic diversity (Fig. 2). ELSS, *Miscanthus* spp., and *S. spontaneum* each showed distinct separation from the other species. *Saccharum robustum, S. officinarum,* hybrid cultivars, and *S. edule* grouped together in a major cluster with subclusters concentrated independently with *S. robustum* and hybrid cultivars. *Saccharum sinense* and *S. barberi* also showed distinction, but overall had little intra-species diversity. *Miscanthus* spp. were most distant from other clones followed by ELSS and *S. spontaneum*.

**Figure 3:**
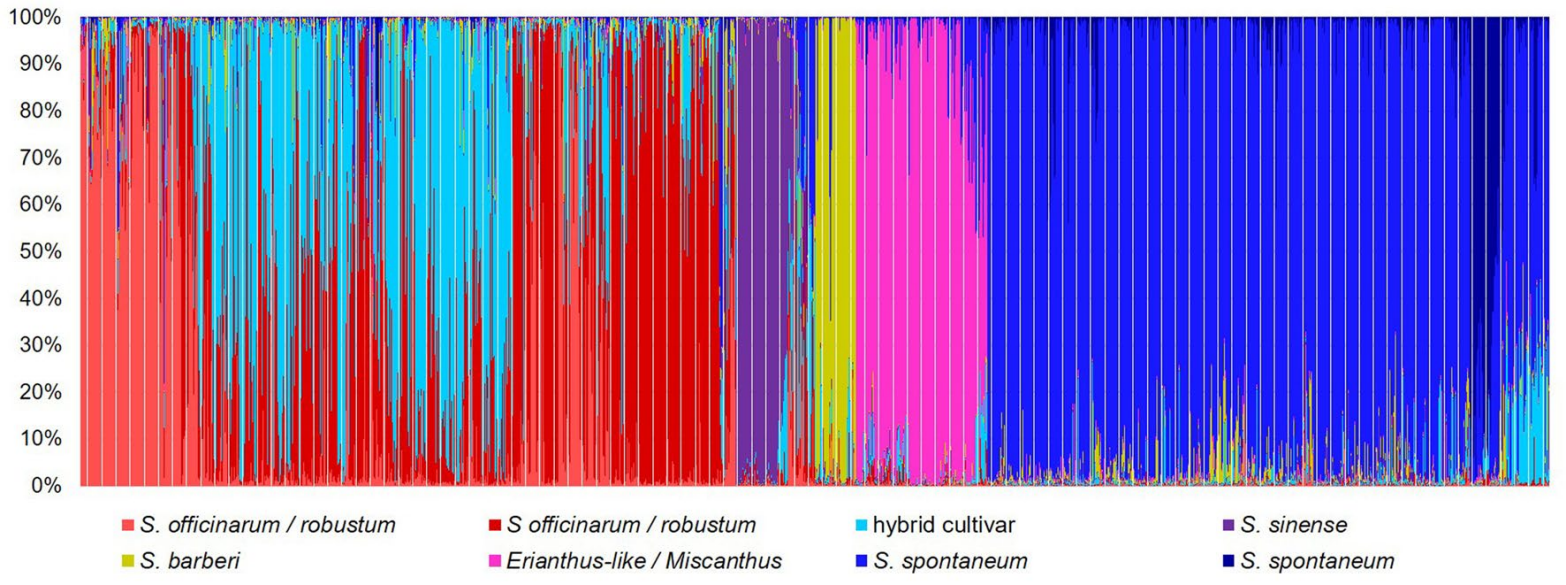
Population structure of 1,485 clones (horizontal axis) showing eight sub-populations. Species groups *Saccharum officianarum / robustum, and S. spontaneum* were each represented by two groups. Clones are in the order of the clones in the neighbor joining analysis for comparison (Fig. 2). *Erianthus-*like *Saccharum* spp. included *S. arundinaceum*, *S. bengalense*, *S. ravennae*, *S. rufipilum*, *S. brevibarbe, S. kanashiroi, S. procerum,* and unknown species previously identified as *Erianthus.* The values in the vertical axis represent the likelihood in percent of an individual belonging to one of the eight colored sub-populations.

The eight sub-populations delineated by the structure analysis had near direct correspondence to species groups (Fig. 3). The first two sub-populations corresponded to both *S. officinarum* and *S. robustum* with no clear distinction between them. Other sub–populations corresponded to hybrid cultivars, *S. sinense,* and *S. barberi.* ELSS and *Miscanthus* spp. comprised one sub-population, whereas *S. spontaneum* was delineated by two sub-populations.

**Figure 4:**
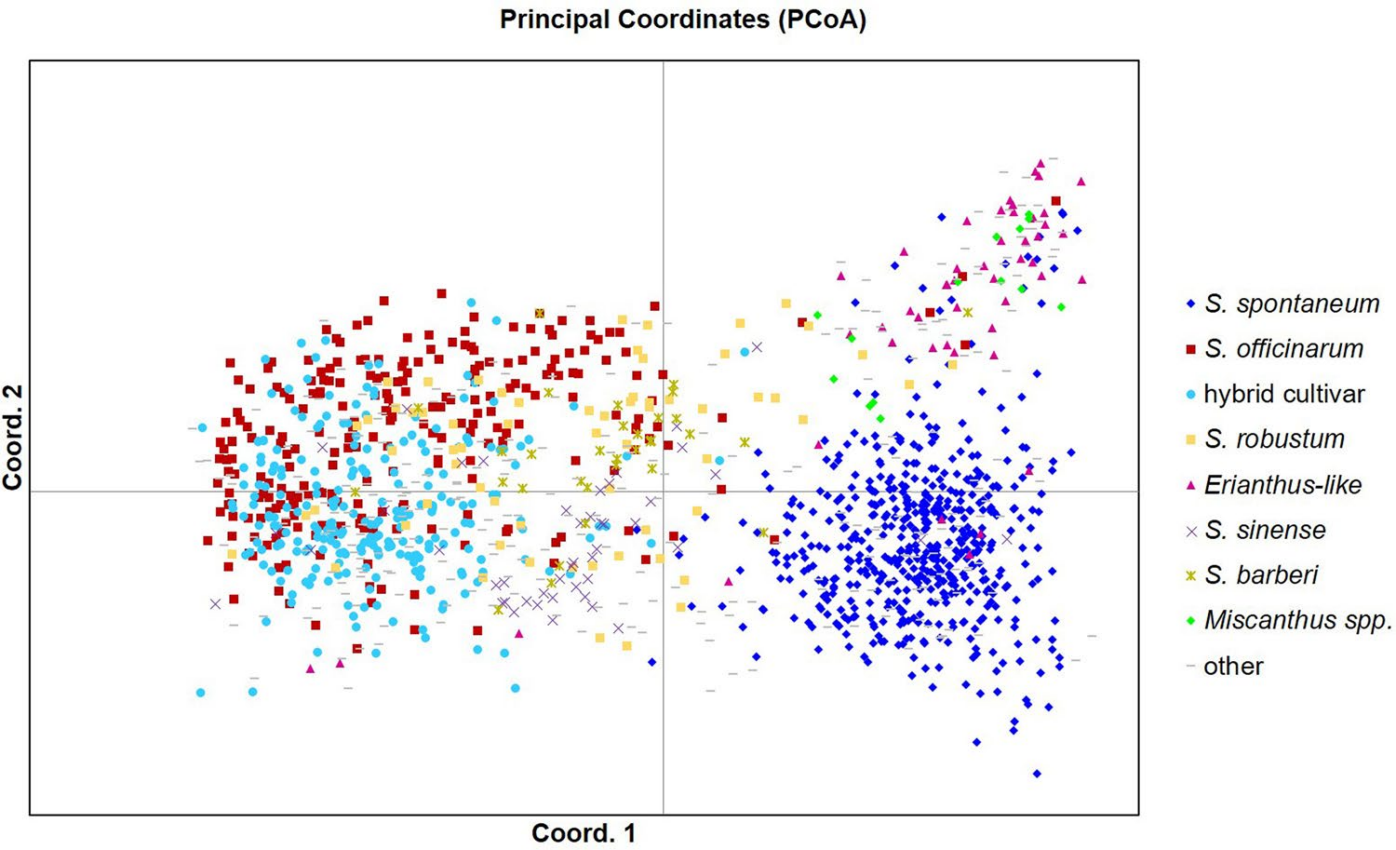
Principal coordinate analysis (PCoA) of 1,485 clones showing inter- and intra-species differentiation. *Erianthus-*like *Saccharum* spp. included *S. arundinaceum*, *S. bengalense*, *S. ravennae*, *S. rufipilum*, *S. brevibarbe, S. kanashiroi, S. procerum,* and unknown species previously identified as *Erianthus.* Other species included *Coix lacryma-jobi, Imperata* sp., *Sorghum polumosum, Saccharum edule,* and unknown.

Principal coordinate analysis (PCoA) showed definite variance between species groups (Fig. 4). The coordinates cumulatively accounted for 12.8% of the total variation, with the first three accounting for 8.04, 2.55, and 2.21%, respectively. *Saccharum spontaneum* grouped by itself with some outliers that grouped with the cluster comprising ELSS and *Miscanthus* spp. *Saccharum officinarum, S. robustum,* and hybrid cultivars grouped together but with distinct centroids. *Saccharum sinense* and *S. barberi* formed separate clusters that were close to each other bordering the clusters of the *S. officinarum* and *S. robustum* clones and between the *S. spontaneum* and *S. officinarum* and *S. officinarum*/ *S. robustum*/ hybrid cultivars cluster.

AMOVA was conducted on the 1,485 clones with the sub-populations derived from three independent diversity analysis-based divisions (Supplementary Table S5). The first division was based on the clones with nine groups consisting of *S. spontaneum, S. officinarum,* hybrid cultivars*, S. robustum,* ELSS*, S. sinense, S. barberi, Miscanthus,* and others, which included *Coix lacryma-jobi, Imperata* sp*., Sorghum polumosum, Saccharum edule*, and unknown species (Fig. 1). The second division was based on the DARwin neighbor-joining on Dice dissimilarities. Here clones grouping together were considered part of the same species groups. This eliminated the ‘other’ group and combined *S. officinarum* and *S. robustum* leaving seven groups. Clones falling in the *S. officinarum / S. robustum /* hybrid cultivar complex that were not hybrid cultivars were considered part of the *S. officinarum / S. robustum* group. The third grouping was based on STRUCTURE model estimates using the group that was estimated to contribute the largest proportion to the genome. All three AMOVA showed significant differences within sub-populations with *p* values ≤ 0.001 (Supplementary Table S5). Genetic variation within a sub-population was high (85.1–90.7%), whereas 9.3, 14.4, and 14.9% of the variance was attributable to between sub-population variance in the name-based division (groups by species), the DARwin and the STRUCTURE divisions, respectively.

### Sugarcane diversity panel (SDP1) selection

**Figure 5:**
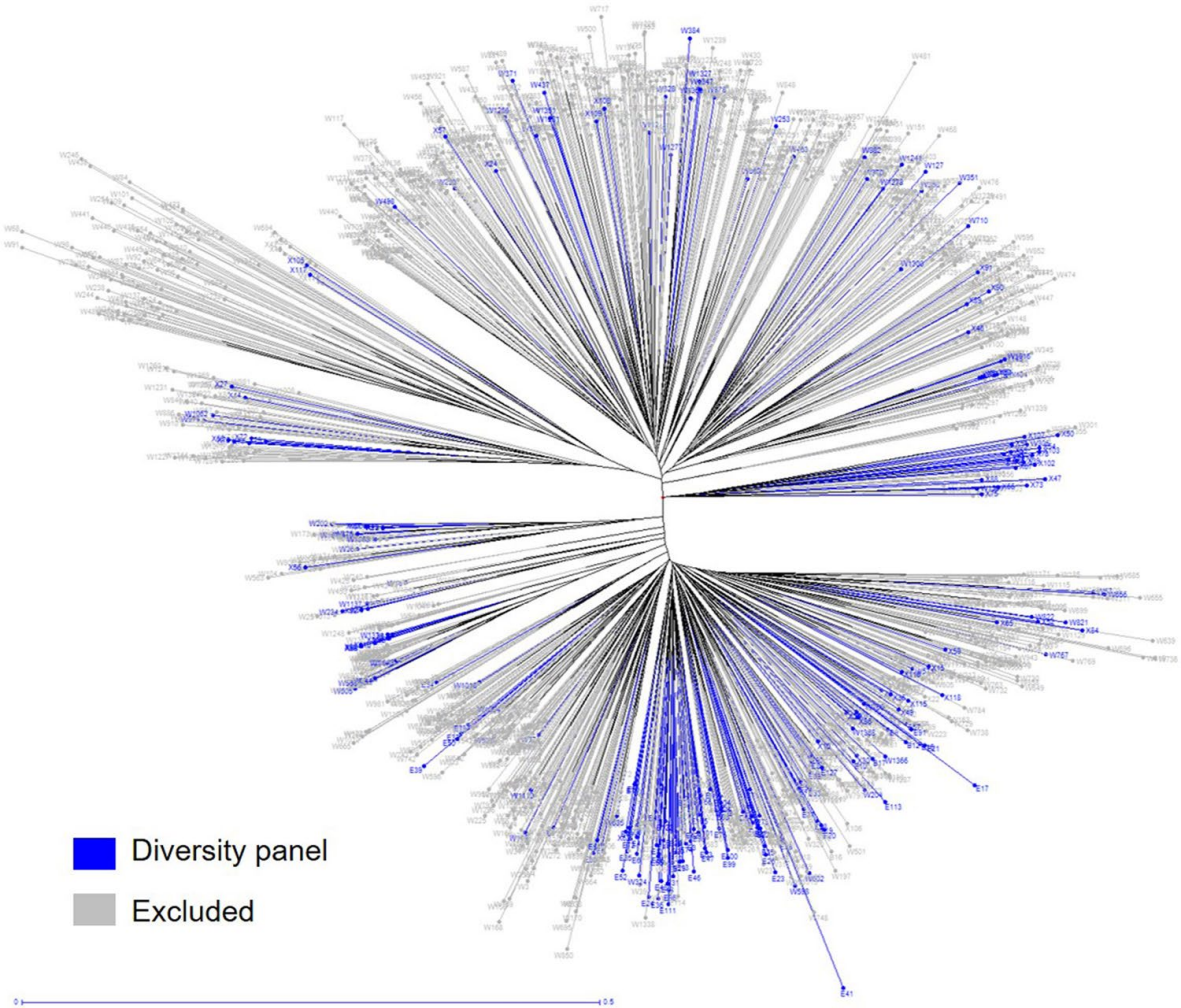
Neighbor-joining tree showing 309-clone diversity panel. Two-hundred thirty-eight clones (blue) were selected based on maximum length subtree program of DARwin with representation from different subclusters. Fifty-seven Louisiana commercial cultivars and 14 F_1_ clones from basic base broadening program were included to maximize representation of Louisiana clones and minimize representation of *Saccharum spontaneum*.

A 309-clone diversity panel designated as SDP1 was selected by the combination of the molecular markers-based genetic diversity using the maximum length subtree algorithm in DARwin (238 clones) and Louisiana sugarcane breeders selection wherein clones (57 Louisiana commercials and 14 basic F_1_ progeny) were picked from different subclusters maximizing the clones existent in Louisiana to minimize the number of clones to be imported from the WCSRG. SDP1 consisted of 284 clones from Louisiana that represent clusters of clones within WCSRG and Louisiana breeding programs. The remaining 25 clones require importation from the WCSRG. Over 100 of the SDP1 clones were Louisiana hybrid cultivars that consisted of both historic and current clones. Louisiana clones exhibited some clustering, but were fairly well dispersed across the entire study population (Fig. 5). *Saccharum spontaneum*, being a noxious weed, cannot be grown in the field making accurate phenotyping of traits more difficult. Therefore, *S. spontaneum* clones’ representation in the SDP1 were intentionally minimized where hybrids x *S. spontaneum* F_1_s developed by the base-broadening introgression breeding program of the USDA-ARS replaced 31 *S. spontaneum* clones*.* Representation of the ELSS and *Miscanthus* spp. from the WCSRG were intentionally kept to a minimum. The remainder of SDP1 was proportionally distributed over other species groups (Fig. 1).

The gene diversity (h) for different species groups were comparable, ranging from 0.13 for *Erianthus-like* species to 0.15 for hybrids (Supplementary Table S3). The overall average gene diversity of all species groups was lower (0.16). The polymorphic alleles were highest for *S. spontaneum* (88%) followed by 77.8% for hybrids. *Erianthus-like* species had lowest allele polymorphism (40.2%).

Nei’s genetic distance (D) showed that *Erianthus-like* group was the most genetically distant from other species (0.029–0.041) with the highest D (0.041) with *S. robustum*. Expectedly, D (0.007) was the smallest between hybrid cultivars group and *S. officinarum*, followed by the equal D (0.016) between *S. officinarum* and *S. robustum* and between *S. officinarum* and *S. barberi* (Supplementary Table S4). *Saccharum sinense* and *S. barberi* were genetically close at D = 0.018.

Analysis of variance showed significant differences within sub-populations (Supplementary Table S5) with high genetic variation among the clones within a sub-population (83.0–89%), whereas 11–17% variation was attributed to sub-populations by species groups, and DARwin and the STRUCTURE defined divisions.

### Genetic diversity study of the mini-core

SSR search of the cold stress responsive genes in *Sorghum* identified 93 different kinds of SSR motifs. Trinucelotide repeats were the highest (55%), of which the GCC/GGC motif had the highest frequency (22), followed by tetra (24%), hexa (10%), penta (6%), and di (5%) (Supplementary Fig. S1). Of the 100 SSR primer pairs used to genotype the mini-core, 67 were polymorphic producing a total of 1,377 alleles with 37.8 and 27.8 as the average and effective number of alleles, respectively (Supplementary Table S6). Polymorphism information content (Nei's gene diversity, h) of the markers ranged between 0.005 and 0.484 with a mean of 0.211 (Supplementary Table S6).

**Figure 6:**
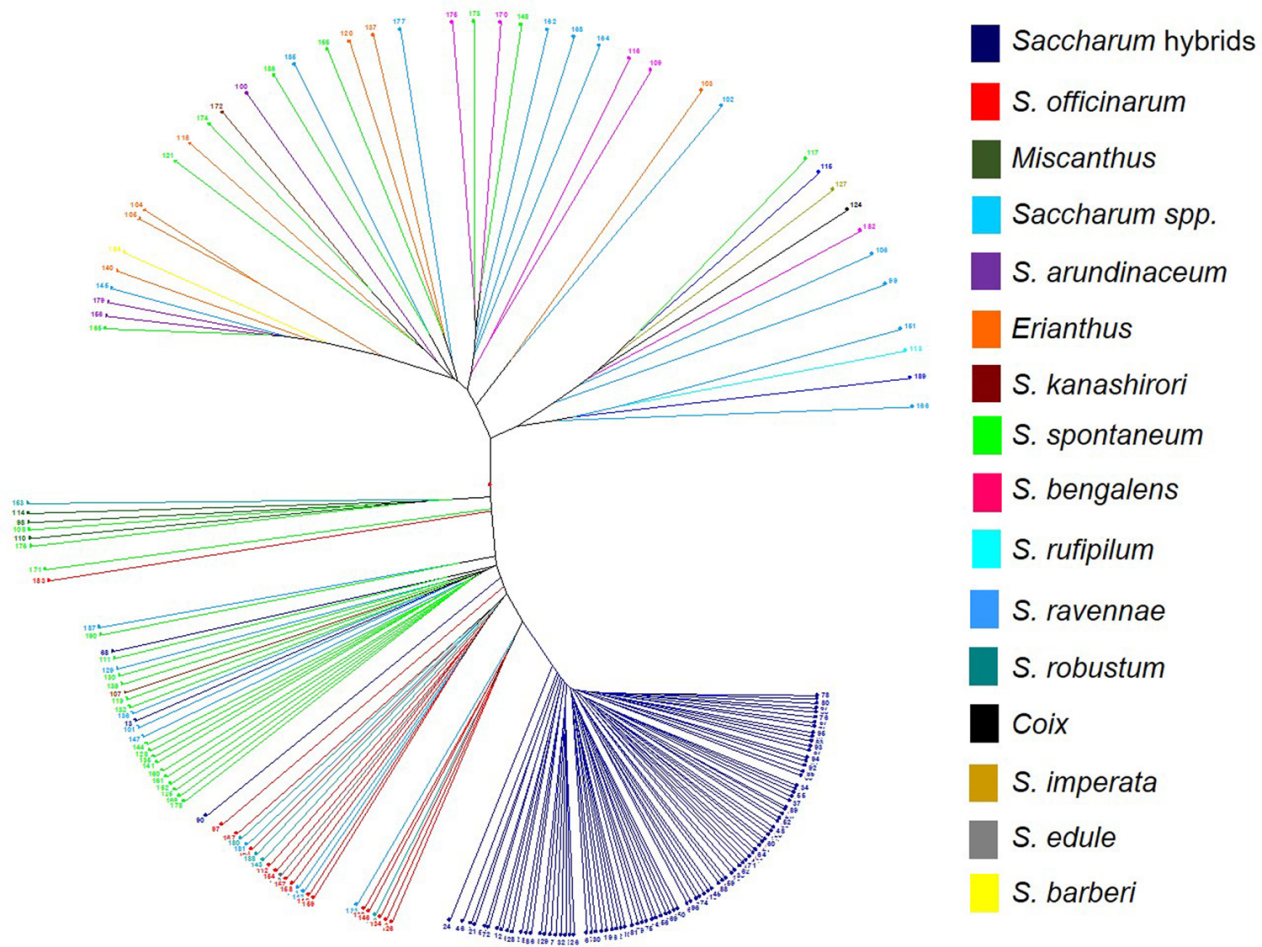
Dendrogram generated with an unweighted pair group method analysis (UPGMA) of the 190-clone sugarcane mini-core based on cold tolerance gene-derived SSR markers.

**Figure 7:**
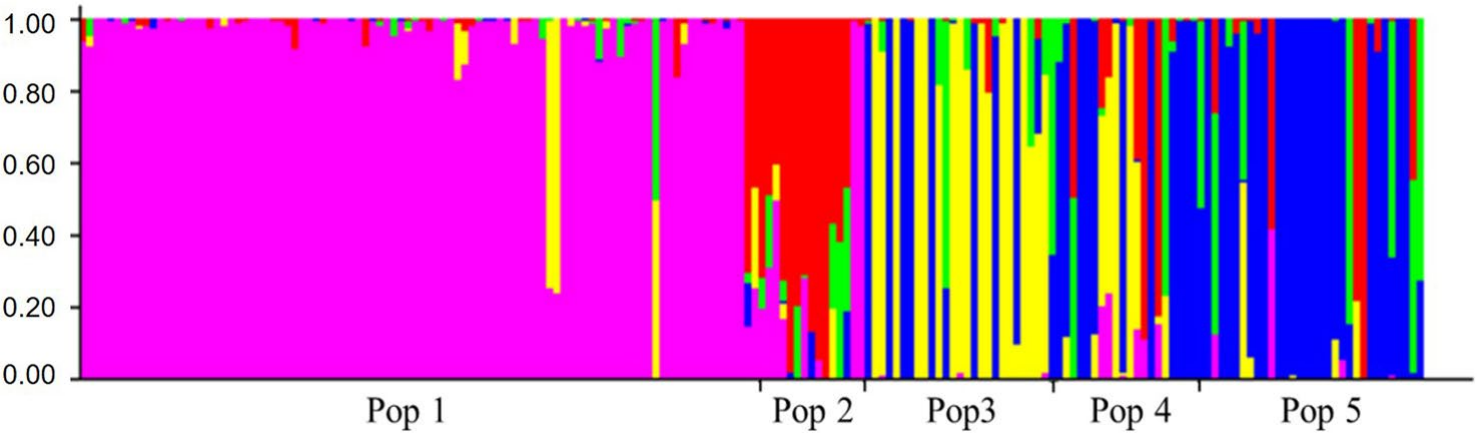
Population structure of the 190-clone sugarcane mini-core using the Structure bar plot (K = 5). Pop 1 = *Saccharum* hybrids, Pop 2 = *S. officinarum*, Pop 3 = *S. spontaneum*, Pop 4 = *Saccharum* sp., Pop 5 = *S. robustum*, *Erianthus*, *Miscanthus*, *S. arundinaceum*, *S. kanashiroi*, *S. ravennae*, *S. rufipilum*, *S. imperata*, *Coix*, *S. edule*, *S. barberi*.

**Figure 8:**
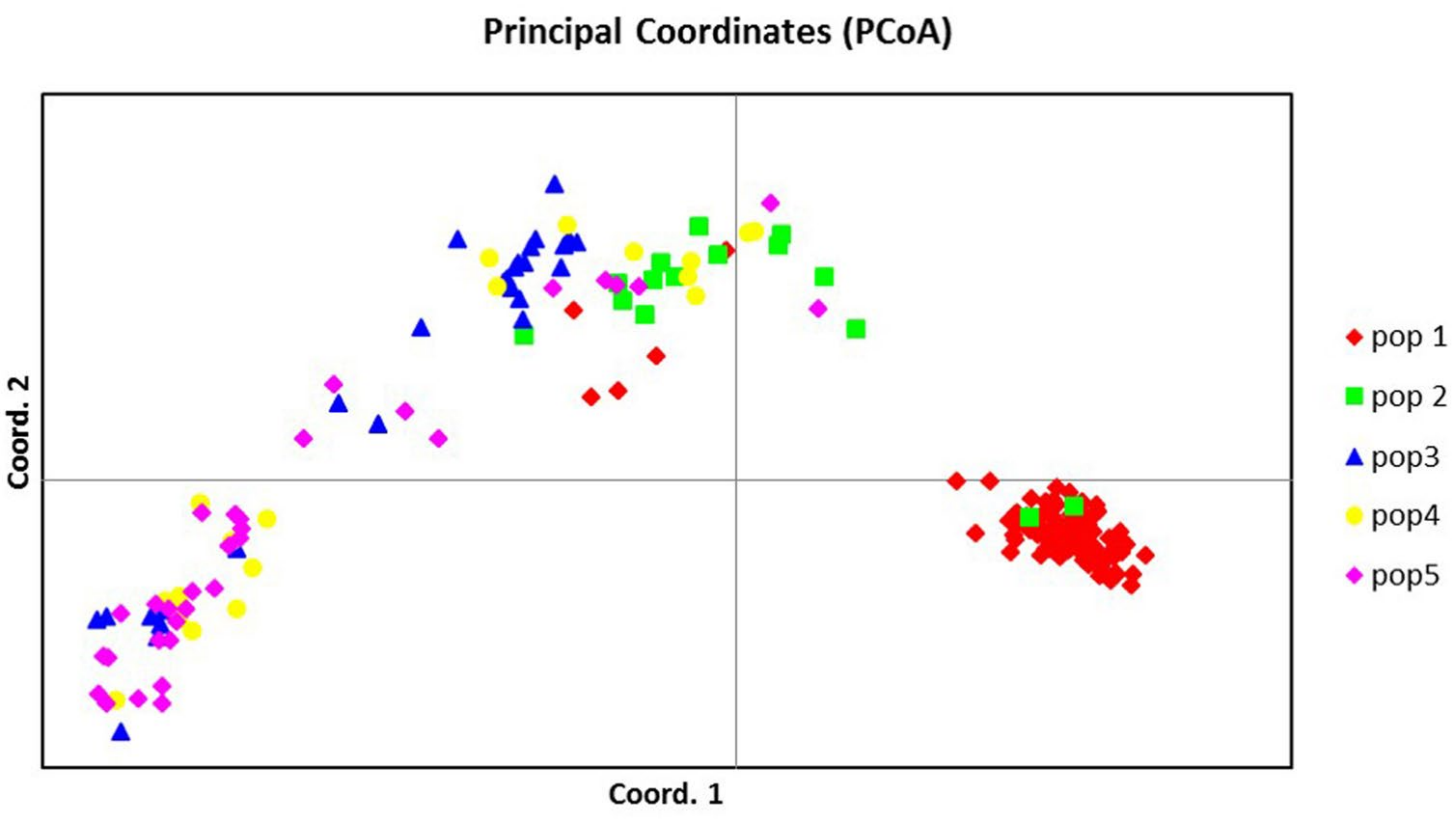
Principal coordinate analysis of the 190-clone sugarcane mini-core. Coordinate 1 = 14.24, Coordinate 2 = 3.87, Coordinate 3 = 1.97. Pop 1: *Saccharum* hybrids, Pop 2: *S. officinarum*, Pop 3: *S. spontaneum*, Pop 4: *Saccharum* sp., Pop 5: *S. robustum*, *Erianthus*, *Miscanthus*, *S. arundinaceum*, *S. kanashiroi*, *S. ravennae*, *S. rufipilum*, *S. imperata*, C*oix*, *S. edule*, *S. barberi*.

The dendrogram generated through unweighted pair group method of arithmetic mean (UPGMA) exhibited three distinct clusters (Fig. 6). Cluster I contained two subclusters, where subcluster IA was predominated by *Saccharum* hybrids and close to the subcluster IB containing mostly *S. officinarum*. *Saccharum spontaneum* populated subcluster IIA, which was close to Cluster IIB with clones listed as unknowns. The third cluster consisted of two subclusters, where *Miscanthus*, *S. edule*, *S. kanashiroi*, and *S. robustum* formed subcluster IIIA, and *S. arundinaceum*, *S. erianthus*, *S. bengalense*, *S. barberi*, *S. imperata*, *S. rufipilum*, *S. coix*, *S. ravennae*, and *S. kanashiroi* grouped together as subcluster IIIB (Fig. 6). On the other hand, Bayesian model of structure grouped the clones into five clusters with Louisiana hybrids in a separate cluster (Fig. 7). The principal coordinate analysis (PCoA) based on Euclidean distance between clones was in agreement with the cluster analysis, and coordinates 1, 2, and 3 explained 14.24%, 3.87%, and 1.97% of the variation, respectively (Fig. 8).

Louisiana hybrid clones had the highest number of unique alleles (37 of the population total of 67) (Supplementary Table S7) when the mini-core was analyzed with the alleles generated by the 11 SSR primers used for the entire 1,485 clones. On the other hand, the population group containing *Miscanthus* and *Erianthus* had the highest number of unique alleles when analyzed with cold-responsive genes-derived SSR primer pairs (Supplementary Table S7). The mean diversity among the populations with cold responsive genes-derived SSRs (0.218) was higher than that with 11 SSRs (0.182) (Supplementary Table S7). Hierarchical analysis of molecular variance (AMOVA) showed equal amount of variation among and within populations at 19% and 81%, respectively with both cold-responsive genes-derived SSRs and 11 SSRs used for the entire population of 1,485 clones (Supplementary Table S8). Nei’s genetic identity between populations was 0.900–0.984 (Supplementary Table S9) with cold-responsive genes-derived SSRs compared to 0.942–0.981 with the SSRs used for entire population of 1,485 clones. The average gene diversity in the mini-core (H at 0.156) and SDP1 (H at 0.163) with the alleles generated with 11 SSR primer pairs were comparable.

## Discussion

The clones within WCSRG are genetically diverse and possess desirable alleles for several agronomic traits^35,51^. However, the daunting task of exploiting the potential of a large number of highly genetically complex clones possessing many undesirable alleles has led to the use of only a few clones in sugarcane breeding programs worldwide. The advent of molecular genomic tools and development/utilization of MAS may allow the tremendous potential value and utility of WCSRG to be realized. A core collection with a smaller number of non-redundant clones, representative of the maximum genetic diversity of the entire collection, will facilitate its utilization in commercial breeding^52^, thus effectively and efficiently managing resources on a smaller but diverse population while maximizing genetic gain. To this end, the present study developed a sugarcane diversity panel (SDP1), a core collection selected from 1,485 clones enriched with clones from Louisiana, based on the diversity analysis and breeders’ input (Figs. 1 and 5).

Subtropical and temperate climate specific sugarcane genotypes were not well represented in other core collections previously developed^33–35,53^ thereby limiting their application for GWAS in Louisiana. For instance, the 300-clone core collection developed^35^ includes 228 wild *S. spontaneum*, other *S.* spp., other genera, and unknown species, which potentially need to be maintained in pots on concrete. This could hinder the phenotypic characterization of the panel under natural field conditions. SDP1, enriched with Louisiana clones, is expected to circumvent this problem as it contains less than 50 wild-type clones and all of these clones are already maintained at the USDA Sugarcane Research Unit in Houma, Louisiana.

While it is challenging to accomplish very good coverage of the complex *Saccharum* spp. genome with only 11 primers as used in the present study, the random coverage of the alleles over the genome was possible due to the multiallelic nature of the SSR primers^54^ distributed over the 10 sugarcane homeologous groups. The 11 SSR primers generated a total of 423 alleles with an average of ~ 38.45 alleles per locus. The range (0.172 to 0.375) of the PIC values of the SSR primers used in the entire collection was similar to the range (0.195 to 0.375) reported earlier^35^. Previous studies have used nearly two times fewer total alleles and average number of alleles per SSR for diversity analysis, such as 205 alleles with 13.67 per locus^55^, 209 alleles with 5.8 per locus^35^, and 261 alleles with 7.35 per locus^56^. Thus, 423 alleles were considered enough to randomly capture a significant proportion of the genetic diversity within the *Saccharum* genome to effectively discriminate accessions among different species groups. This was evident from the bootstrap results (Supplementary Table S10) that showed that 423 polymorphic alleles reduced the CV to 2.3% and 114 alleles were sufficient enough to result in the recommended 5% coefficient of variation (CV)^57,58^.

The clusters generated by neighbor-joining analysis, structure analysis, and PCoA were similar. The only difference was that, unlike the neighbor-joining analysis, neither the structure analysis nor the PCoA produced a *Miscanthus* spp. cluster separate from the ELSS. The species groups described by neighbor-joining were *S. spontaneum, S. officinarum / robustum,* hybrid cultivars, ELSS, *S. sinense, S. barberi,* and *Miscanthus* spp. *Saccharum* experienced one genome duplication shared with *Miscanthus*, but an additional, more recent lineage-specific duplication leading to autopolyploidization in *Saccharum* species may explain the distance between *Saccharum* hybrids and *Miscanthus*^59^. Principal coordinate analysis showed hybrid cultivars and *S. officinarum / robustum* clustering together*.* Additionally, the PCoA most likely could provide visualization for the majority of the variance between populations with 10.59% of the total variance explained by the first two coordinates, which was close to 9.3, 14.4, and 14.9% found by the AMOVA between groups. All this evidence supports the current *Saccharum* species delineations.

The low robustness of the neighbor-joining tree was due to the combination of varying numbers of clones within a species in the population and the use of minor alleles. Because of the nature of the population, alleles primarily present in *S. spontaneum,* in particular, or in *S. robustum*, *S. officinarum,* or hybrid cultivars required little representation within their primary species to be included at higher frequencies under bootstrapping, while alleles primarily present in species with less clones needed more representation. The cut-off allele frequency of 0.01 used in this study affected 15 clones, represented by 3% of the *S. spontaneum*, but up to 107% of the *Miscanthus* spp. clones. Thus, each species did not have equal representation among the alleles used, which resulted in changes in the diversity-based tree when different samples were selected. Nevertheless, the genetic diversity was well represented, as demonstrated by the neighbor-joining, structure and principal coordinate analyses.

Results of the diversity analysis of the WCSRG from the present study and that of Nayak et al.^35^ were comparable. In the present study, overall averages were 0.213 and 0.338 for gene diversity and Shannon’s information indexes, respectively, as compared to 0.310 and 0.438, respectively, observed by Nayak et al.^35^. The percentage of polymorphic alleles in the present study ranged from 54.37 to 99.29, whereas it ranged between 75.60 and 99.52 for Nayak et al.^35^. The genetic distance between groups/species in the present study was lower than previously published reports. For example, the distance between *S. spontaneum* and *S. officinarum* was 0.55 as compared to 0.79 reported by Nayak et al.^35^. The low averages in the present study could be due to the retention of minor alleles. Nevertheless, *S. spontaneum* remained the most genetically diverse species, primarily due to its high variation in chromosome number (2n = 40–128), diversity of habitats, and widest geographic distribution^43,60^.

Clustering analysis results of the WCSRG and Louisiana collection in the present study were similar to that observed by Nayak et al.^35^ where three main clusters: 1) *S. spontaneum*; 2) *S. officinarum,* hybrid cultivars, *S. robustum, S. edule, S. barberi,* and *S. sinense*; and 3) other species were described. However, species within each cluster were resolved better in our study, where seven clusters in the neighbor-joining analysis (*S. spontaneum, S. officinarum / robustum,* hybrid cultivar, ELSS, *S. sinense, S. barberi,* and *Miscanthus* spp.) and six clusters with the structure analysis and the PCoA (*S. spontaneum, S. officinarum / robustum,* hybrid cultivar, ELSS */ Miscanthus* spp., *S. sinense,* and *S. barberi*) were identified. Nayak et al.^35^ left *Erianthus* and *Saccharum* nomenclatures as found in the WCSRG database. They used non-*Saccharum* species as a group, grouping *Erianthus* spp. with *Miscanthus* spp. Considering that several *Erianthus* spp. have been reclassified under *Saccharum*, it is difficult to comprehend if their analysis found the same distinction that was observed in the present study between ELSS and *Miscanthus* spp. The distinction between *Saccharum* spp. and *Erianthus*-like *Saccharum* spp., as found in our study, was also observed earlier^61^.

Genic SSRs derived from the cold-responsive transcripts with a putative function are useful for functional diversity assay in natural populations or germplasm collections. The low polymorphism information content of the genic SSRs (0.211) we observed among the clones in WCSRG was similar to that with a set of cold-responsive genes-derived markers among the Louisiana clones in our previous study^39^. The low polymorphism of genic SSRs could be explained by the conserved sequence of the transcribed regions within the *Saccharum* genus^62^ or by the low transmission of diverged sequence differences between the *Saccharum* spp.^51^. Genetic similarity was high among the sugarcane hybrids, which is likely because only a few parental clones were involved in the development of the foundation clones through nobilization in breeding programs^63^. Alleles at these loci are possibly fixed as a result of a limited number of founder parents and subsequent selection. The Louisiana sugarcane hybrids formed a distinct group, close to the group containing *S. officinarum* clones, which is expected because most of the modern cultivars inherited the major part (~ 80–85%) of the genome of *S. officinarum* during nobilization events^64^. Besides, Louisiana-bred clones grouped in a subcluster within the hybrids cluster, which may be because they were developed in subtropical conditions under selection pressure from cold temperatures. Similar to the *S. spontaneum* in the entire collection of 1,485 clones, *S. spontaneum* clones in subcluster 3 of the mini-core were close to unknown *S.* spp. clones in cluster 4. This supported our assumption that most, if not all, of the unknown clones in the WCSRG are *S. spontaneum*. The non-correspondence of genetic similarity of clones within a species group within WCSRG and the mini-core is believed to be due to the varied cold stress response of the clones. This is justified by the high percentage (81.2%) of variation within a species group (population) compared to 18.8% among the species groups (Supplementary Table S8) and high average gene diversity (0.218) in the mini-core (Supplementary Table S7) as compared to the 0.208 in the present study (Supplementary Table S3) and 0.304^35^ within the WCSRG. The number of alleles unique to the different species groups (average 17.4; Supplementary Table S7) suggested a wide genetic variation in cold stress response of the mini-core clones. Ongoing studies are focused to elucidate the role of variation in microsatellite loci in discriminating clones with varied cold tolerance and future development of functional markers for their use in MAS in breeding for cold tolerant sugarcane cultivars.

Taken together, the genetic diversity analysis based on genotypic data of 1,485 clones helped Louisiana breeders in the selection of 309 clones (20.8%) as the SDP1 that, with an average h at 0.163 (Supplementary Table S3), captured the diversity found in the population (average h at 0.208), including the Louisiana core collections. SDP1 and mini-core shared 324 and 319 alleles, respectively out of 423 alleles in the entire collection of 1,485 clones (Supplementary Table S2). The percentage of variation among the populations in the entire collection and SDP1 were comparable (Supplementary Table S5). The genetic diversity of the mini-core by cold-responsive genes-derived SSR markers, which potentially affected the function of the cold-responsive proteins, suggested the diversity present for phenotypic traits, in this case cold response, within SDP1. SDP1 is now being used for phenotyping of various traits of agronomic importance for GWAS studies to identify markers associated with those traits. Phenotypic characterization within SDP1 can then be used to identify clones of special interest for particular traits, and these results will allow more effective introgression of useful alleles from wild/exotic clones in the already existing sugarcane base-broadening program of Louisiana to improve commercial sugarcane or energy cane variety development. In addition, another important value of SDP1 will be to serve as the validation platform for trait-markers identified from QTL mapping studies involving biparental populations. This function will allow the realization of the potential for molecular breeding for improved cultivar development in sugarcane.

## Supplementary information

Supplementary Figure S1.

Supplementary Table S1.

Supplementary Table S2.

Supplementary Table S3.

Supplementary Table S4.

Supplementary Table S5.

Supplementary Table S6.

Supplementary Table S7.

Supplementary Table S8.

Supplementary Table S9.

Supplementary Table S10.
