# Supplementary material for: An enriched sugarcane diversity panel for utilization in genetic improvement of sugarcane
Source: Sci Rep. 2020 Aug 7;10:13390. doi: 10.1038/s41598-020-70292-8 (PMC7414218; doi:10.1038/s41598-020-70292-8)

Supplementary Fig. S1.

An Enriched Sugarcane Diversity Panel for Utilization in Genetic Improvement of Sugarcane

Nathanael D Fickett[1,a,¥], Leila Ebrahimi[1,b,¥], Arnold P Parco[1,c,¥], Andres V Gutierrez[1,¥], Anna L Hale[2], Michael J Pontif[3], James Todd[2], Collins A Kimbeng[3], Jeffrey W. Hoy[3,4], Tomas Ayala-Silva[5], Kenneth A Gravois[3], Niranjan Baisakh[1*]

[1]School of Plant, Environmental and Soil Sciences, Louisiana State University Agricultural Center, Baton Rouge, LA 70803

[2]Sugarcane Research Unit, USDA-ARS, Houma, LA

[3]Sugar Research Station, Louisiana State University Agricultural Center, St. Gabriel, LA

[4]Department of Plant Pathology and Crop Physiology, Louisiana State University Agricultural Center, Baton Rouge, LA

[5]Tropical Agricultural Research Station, Mayaguez, PR

[a]Present address: Vermont Mutual Insurance Group, Montpellier, VT

[b]Present address: University of Tehran, Aburaihan Campus, Iran

[c]Present address: Certis Inc., Baton Rouge, LA

¥ Contributed equally to this work

*Correspondence: Email: nbaisakh@agcenter.lsu.edu; Telephone: +1 225 5781300

Supplementary Fig. S1. Frequency of SSR motifs in the cold-responsive genes of *Sorghum*

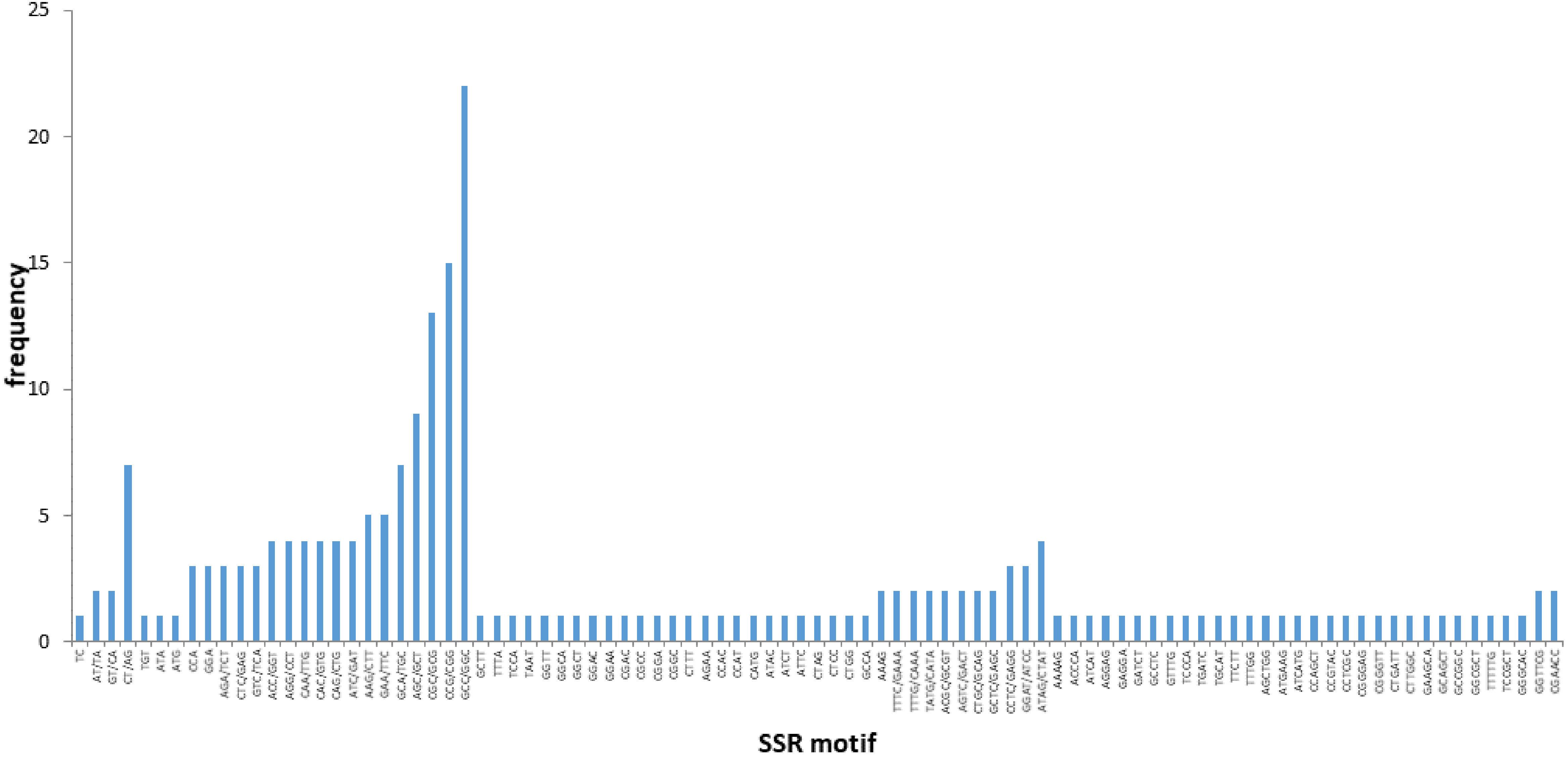

Supplement: Supplementary file 1 — Supplementary Figure S1. [file 41598_2020_70292_MOESM1_ESM.pdf]
